# Integrative Models of Histopathological Image Features and Omics Data Predict Survival in Head and Neck Squamous Cell Carcinoma

**DOI:** 10.3389/fcell.2020.553099

**Published:** 2020-10-29

**Authors:** Hao Zeng, Linyan Chen, Yeqian Huang, Yuling Luo, Xuelei Ma

**Affiliations:** ^1^State Key Laboratory of Biotherapy, Department of Biotherapy, Cancer Center, West China Hospital, Sichuan University Collaborative Innovation Center, Chengdu, China; ^2^West China School of Medicine, West China Hospital, Sichuan University, Chengdu, China

**Keywords:** head and neck cancer, histopathological image, genomics, transcriptomics, proteomics, prognosis

## Abstract

**Background:**

Both histopathological image features and genomics data were associated with survival outcome of cancer patients. However, integrating features of histopathological images, genomics and other omics for improving prognosis prediction has not been reported in head and neck squamous cell carcinoma (HNSCC).

**Methods:**

A dataset of 216 HNSCC patients was derived from the Cancer Genome Atlas (TCGA) with information of clinical characteristics, genetic mutation, RNA sequencing, protein expression and histopathological images. Patients were randomly assigned into training (*n* = 108) or validation (*n* = 108) sets. We extracted 593 quantitative image features, and used random forest algorithm with 10-fold cross-validation to build prognostic models for overall survival (OS) in training set, then compared the area under the time-dependent receiver operating characteristic curve (AUC) in validation set.

**Results:**

In validation set, histopathological image features had significant predictive value for OS (5-year AUC = 0.784). The histopathology + omics models showed better predictive performance than genomics, transcriptomics or proteomics alone. Moreover, the multi-omics model incorporating image features, genomics, transcriptomics and proteomics reached the maximal 1-, 3-, and 5-year AUC of 0.871, 0.908, and 0.929, with most significant survival difference (*HR* = 10.66, 95%CI: 5.06–26.8, *p* < 0.001). Decision curve analysis also revealed a better net benefit of multi-omics model.

**Conclusion:**

The histopathological images could provide complementary features to improve prognostic performance for HNSCC patients. The integrative model of histopathological image features and omics data might serve as an effective tool for survival prediction and risk stratification in clinical practice.

## Introduction

Head and neck cancer (HNC) comprises a variety of carcinomas that originate from head and neck region, including the nasal cavities and sinuses, oropharyngeal cavities, larynx, major and minor salivary glands (Lydiatt et al., 2017). Moreover, HNC is the sixth most common cancer with yearly incidence of 500,000–600,000 cases worldwide (Suh et al., 2014). The head and neck squamous cell carcinoma (HNSCC) accounts for more than 90% of the cases (Suh et al., 2014). Tobacco, alcohol consumption and human papillomavirus (HPV) infection are common risk factors related with cancer incidence (Chaturvedi et al., 2008; Hashibe et al., 2009). The 5-year mortality rate has remained flat at around 50% and not been improved by significant progress in treatment regime (Chiesa et al., 2016). Prognosis prediction represents a good opportunity to improve patient survival, because prognostic markers contribute to the risk stratification and individualized treatment protocol. Only traditional clinical predictors such as tumor stage and tumor depth are unable to meet the growing demand of precision oncology (Biankin et al., 2015). Therefore, it is of crucial importance to apply more effective prognostic markers and models for patients with HNSCC.

The histopathological images obtained by biopsy or resection of lesions are widely used in the definitive diagnosis, staging and prognosis of cancer patients. In recent years, the computer-aided images analysis systems have been applied to assess digital pathological images, with the advantages of high accuracy, rapidity and consistency, which can make up for the shortage of manual evaluation (Zhang et al., 2015). The extracted histopathological image features (HIF) encompass multiple morphological and histological information, such as cell shape, size, texture patterns of nuclei and cytoplasm (Soliman, 2015). Although these features cannot be recognized by pathologists with visual inspection, previous studies have shown the significant prognostic value of HIF in several cancers, including breast cancer, lung cancer and brain tumor (Sertel et al., 2009; Kong et al., 2013; Wang et al., 2013; Chen et al., 2015; Yu et al., 2016).

In addition to pathological images, other omics profiles including genomics, transcriptomics and proteomics have also been widely used for risk stratification and survival prediction of cancer patients (Wallner et al., 2006; Yanaihara et al., 2006). For example, enhanced TP53 mutation, gene duplication and 3p loss were found in recurrent and metastatic HNSCC with primary HPV infection, while TERT promoter mutation was more frequent in HPV-negative cohort (Morris et al., 2017). The TRAF3 deletion, E2F1 amplification and PIK3CA mutation were related with abnormal activation of NF-κB signaling and other carcinogenic pathways in HPV-positive HNSCC (Cancer Genome Atlas Network, 2015). Moreover, the TP53 gain-of-function (GOF) variant and mTOR pathway activation were predictive of worse survival and early progression in HPV-negative HNSCC patients (Niehr et al., 2018).

However, given the heterogeneity of cancer patients and complexity of survival prediction, the research work is far from stopping. The interconnections between histopathology and omics and how to integrate these features for better outcome prediction and personalized treatments still need further exploration. Previous study has revealed a significant association between several gene expressions (such as HYAL2 and HLA-DRA) and morphological features of nuclei texture in liver hepatocellular carcinoma (Zhong et al., 2019). Also, the obvious interconnections of TP53 mutation and histological features of nuclei and cytoplasm were reported in lung adenocarcinoma (Yu et al., 2017). In addition, some studies established integrative models based on omics and histopathological image data in liver cancer, lung cancer, renal cancer and breast cancer, which showed an improved prognostic accuracy than individual factors (Cheng et al., 2017; Yu et al., 2017; Sun et al., 2018; Zhong et al., 2019). These results indicated the widespread application value and development prospects of histopathological images and omics data for predicting prognosis.

After literature review, we were of opinion that there is still room for improvement. Some studies only focused on single omics profile such as genomics, transcriptomics or proteomics, and lacked the comprehensive survival analyses of each omics combined with histopathological image features. Moreover, the prognostic performance of models that integrate histopathological and omics features for HNSCC patients is still unclear. Therefore, in this study, we aimed to evaluate and compare the prognostic role of histopathological images, genomics, transcriptomics and proteomics in HNSCC patients. Furthermore, different combinations of multi-omics models were established to improve prognostic accuracy, and to highlight the contribution of histopathological images in prognosis modeling.

## Materials and Methods

**FIGURE 1:**
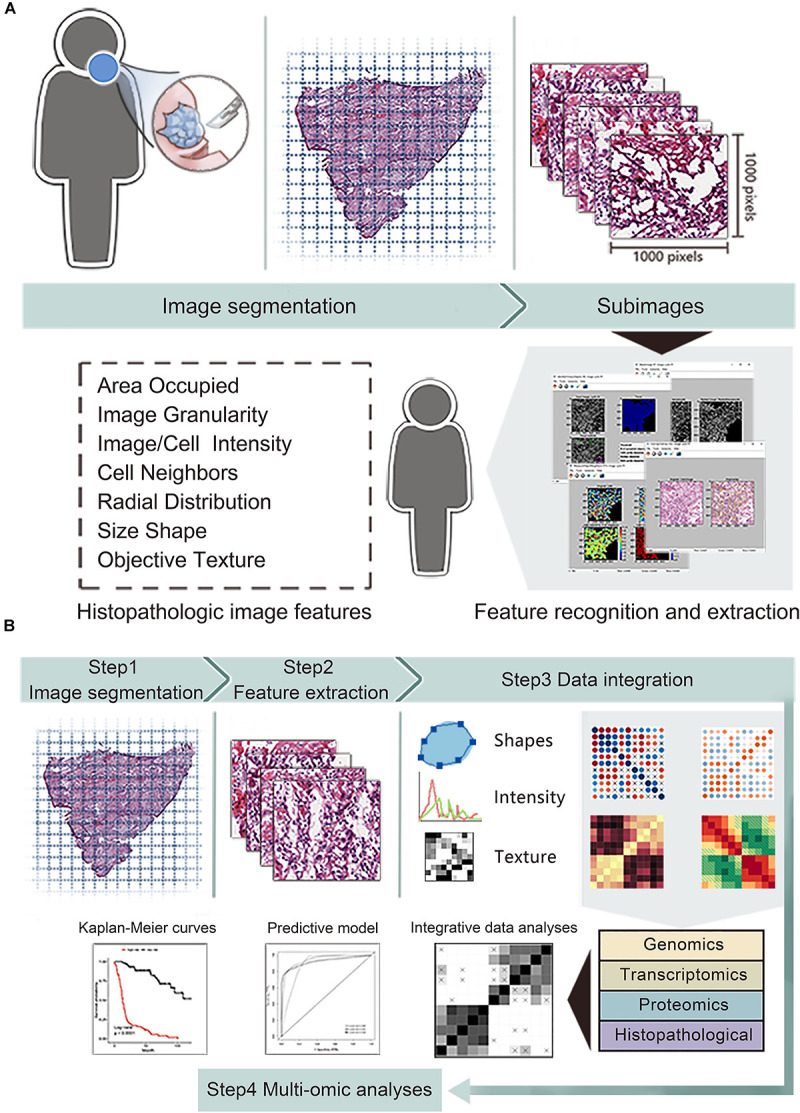
The workflow of data analysis and integration. **(A)** The whole-slide histopathological images of head and neck squamous cell carcinoma were cropped into small sub-images of 1,000 × 1,000 pixels. Then we excluded the sub-images containing white space more than 50% and selected 20 sub-images for each patient. Next, CellProfiler estimated the images and obtained mean value of image features related to shape, intensity and texture. **(B)** We integrated features of histopathological images, genomics, transcriptomics and proteomics to generate improved prognostic models by random forest method with in training set, then evaluated the model predictive performance in validation set.

The overall flowchart of image features extraction and multi-omics prognostic models establishment was presented in Figure 1. The histopathological images were divided into small sub-images and analyzed by CellProfiler to extract image features. Afterward, the random forest (RF) algorithm was used to combine images features and omics to build prognostic models and generate the average prediction accuracy. Finally, we applied time-dependent receiver operating characteristic curve, Kaplan-Meier survival curve, and decision curve analysis to estimate and compare the prognostic values between models. The details of each part were described in the following sections.

### Data Acquisition and Images Segmentation

We obtained a dataset consisting of clinical, genetic and transcriptomics information of HNSCC patients from the Cancer Genome Atlas (TCGA) data portal^1^. The corresponding protein profile via reverse phase protein array (RPPA) were downloaded in the Cancer Proteome Atlas (TCPA) repository^2^. The corresponding hematoxylin and eosin (H&E) histopathological images were downloaded from the Cancer Imaging Archive (TCIA) portal^3^. Since the whole-slide images (20× or 40× magnification) were too large to extract features, we performed images segmentation by the Openslide Python library (Goode et al., 2013) to facilitate subsequent analyses. Firstly, 216 whole-slide images were divided into 341,649 small sub-images of 1,000 × 1,000 pixels, and changed into tiff format from svs format. Next, we excluded the sub-images containing white space more than 50%. Moreover, for each patient, 20 sub-images were randomly included to reduce sample selection bias and decrease calculation amount.

### Histopathological Image Features Extraction

We used CellProfiler^4^ to automatically measure images and extract histopathological features (Carpenter et al., 2006). The images processing and measurement modules of CellProfiler transformed the color images stained by hematoxylin and eosin into grayscale images, then obtained 10 aspects of image features including area occupied, correlation, granularity, image intensity, image quality, object intensity, object neighbors, object radial distribution, object size shape and texture. These features focus on objective image information, which are different from accustomed pathological characteristics (e.g., cellular pleomorphism, nuclear atypia, and mitoses) recognized by visual inspection of pathologists. For instance, object size shape outputs several cell-level features containing area, perimeter, form factor (4πarea/perimeter^2^), eccentricity, lengths of major axis and minor axis, Euler number, Zernike shape features and so on. Texture evaluation module of CellProfiler provides information about variations in the spatial distribution of intensities of grayscale images, including Haralick’s features and Gabor “wavelet” features (Haralick et al., 1973). Image intensity and object intensity describe total pixel intensities in images or specific objects (e.g., nuclei or cells), respectively. Finally, we extracted 593 quantitative image features for each sub-image, then calculated the average values of 20 sub-images for each patient.

### Statistical Analysis

1. Survival analysis: Patients were divided into two groups based on the median value of each histopathological image feature. The hazard ratio (HR) and 95% confidence interval (CI) for overall survival (OS) were calculated by univariate Cox regression analysis. The least absolute shrinkage and selection operator (LASSO)-Cox regression method was also utilized to show significant image features (Tibshirani, 1997). Then Kaplan-Meier survival curve and log-rank test compared the differences of survival results between two groups. The *p*-value < 0.05 was regarded as statistically significant.
2. Feature selection: We first randomly divided the HNSCC patients into training (*n* = 108) or validation (*n* = 108) sets. The genomics data contained 14,794 features and the transcriptome data contained 19,754 features. By contrast, histopathological images contained 593 features and the proteomics contained 161 features. Therefore, in the training set, we included all features of histopathological images and proteomics, while conducted preliminary screening of genomics and transcriptomics to reduce their dimensionality. The 100 most common somatic mutations were used for further analyses. Next, we defined patients with a survival time more than 60 months as the long-term survival group, while died patients with a survival time of 1–12 months were considered as the short-term survival group. The R DESeq2 package was used for the normalization and analysis of differently expressed genes (DEGs) between groups in training set. Then 100 most significant DEGs were applied to predict survival. The feature selection could reduce the potential bias caused by large difference in feature numbers among omics, and may reduce potential confounders (e.g., low frequency mutations or non-significant expressed genes).
3. Gene set enrichment analysis: To find the differences of Kyoto Encyclopedia of Genes and Genomes (KEGG) pathways between short-term and long-term survival groups, we used the Gene Set Enrichment Analysis (GSEA) to sort DEGs according to the degree of differential expression, and examined the enriched gene sets in two groups (Subramanian et al., 2005). Statistical significance was defined as *p* < 0.05 or false discovery rate *q* < 0.25.
4. Integrative prognostic models: Each type of data (histopathological image features/HIF, genomics, transcriptomics, and proteomics) and various fusions of multiple features (HIF + genomics, HIF + transcriptomics, HIF + proteomics, HIF + omics) were involved to evaluate and compare the usefulness in prognosis modeling. In the training set, we applied the random forest (RF) method to build prognostic models via R randomForestSRC package (Breiman, 2001; Ishwaran et al., 2014). RF is a widely used machine-learning method in high-dimensional data processing, which can handle thousands of input variables at the same time, evaluate the predictive ability of each feature and exclude uncorrelated ones. At the same time, it can use internal cross-validation to generate unbiased estimation of generalization error and ensure high accuracy. The RF classifier with 1,000 decision trees and 10-fold cross-validation were used in training set. Next, we calculated the area under the curve (AUC) of time-dependent receiver operating characteristic curve (ROC) to verify the performance and robustness of new models in validation set. Moreover, based on the median value of risk score estimated from models, patients of the validation set could be divided into high-risk and low-risk groups. Then we conducted Kaplan-Meier analysis and log-rank test to evaluate the prediction ability of models. Decision curve analysis (DCA) was performed to measure the net benefits of each model based on 5-year OS (Vickers et al., 2008).

## Results

### Patients Characteristics

A dataset of 216 HNSCC patients (154 males and 62 females) with data of histopathological images and other omics from TCGA project was included (Table 1). The median age at initial diagnosis was 62 years (range 19–90 years) of patients. This cohort comprised squamous cell carcinoma of oral cavity (61 tongue, 6 alveolar ridge, 6 buccal mucosa, 25 floor of mouth, 6 hard palate, and 39 non-specific lesions), larynx (57 patients), tonsil (13 patients), and hypopharynx (3 patients). There were 106 patients died (50 in training set and 56 in validation set) during follow-up, the median survival time was 41.8 months (range 3.5–175.1 months) for alive patients and 15.2 months (range 0.1–213.9 months) for died patients. Moreover, 83 patients had tumor progression including locoregional recurrence, distant metastasis and new primary malignancy. Chi-squared analyses and *t*-tests showed no statistically significant differences in age, gender, tumor types, cancers stage, cancer status and survival time between training and validation groups.

**TABLE 1:** Demographic and clinical characteristics of patients.

Characteristic	Total (*n* = 216)	Training set (*n* = 108)	Validation set (*n* = 108)	*P*-value
Age: mean ± SD	62.0 ± 11.9	61.3 ± 12.3	62.6 ± 11.4	0.434
**Gender**				
Male	154 (71.3%)	76 (70.4%)	78 (72.2%)	
Female	62 (28.7%)	32 (29.6%)	30 (27.8%)	0.764
**Anatomic subdivision**				
Oral cavity	143 (71.3%)	68 (63.0%)	75 (69.4%)	
Larynx	57 (26.4%)	33 (30.6%)	24 (22.2%)	
Others	16 (7.4%)	7 (6.5%)	9 (83.3%)	0.365
**Cancer stage**				
I	11 (5.1%)	5 (4.6%)	6 (5.6%)	
II	35 (16.2%)	14 (13.0%)	21 (19.4%)	
III	35 (16.2%)	17 (15.7%)	18 (16.7%)	
IV	135 (62.5%)	72 (66.7%)	63 (58.3%)	0.546
**Cancer status**				
Tumor free	114 (52.8%)	53 (49.1%)	61 (56.5%)	
With tumor	83 (38.4%)	45 (41.7%)	38 (35.2%)	
NA	19 (8.8%)	10 (9.3%)	9 (8.3%)	0.548
Survival time: mean ± SD	36.7 ± 33.6	32.5 ± 28.7	40.9 ± 37.6	0.064

### Prognostic Value of Histopathological Image Features

**FIGURE 2:**
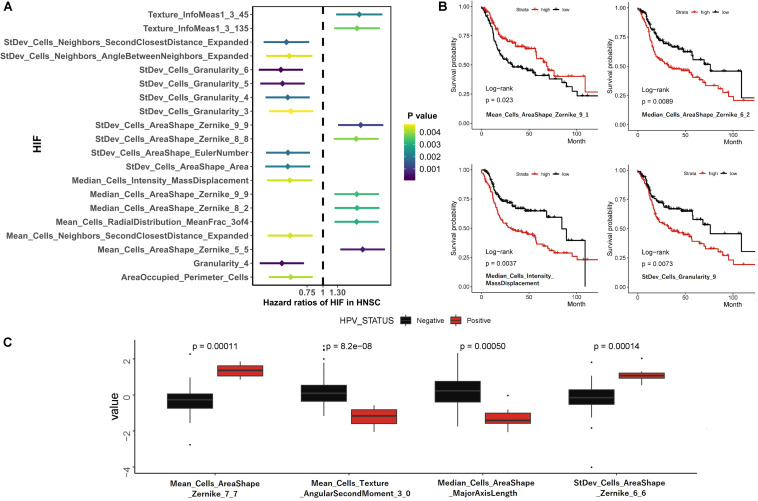
Univariate survival prediction using histopathological image features. **(A)** Survival difference between groups separated by median values of image feature in Cox regression analyses. **(B)** Kaplan-Meier curves for “Mean_Cells_AreaShape_Zernike_9_1,” “Median_Cells_AreaShape_Zernike_6_2,” “Median_Cells_Intensity_MassDisplacement,” and “StDev_Cells_Granularity_9.” **(C)** The different distribution of image features between HPV- and HPV + patients.

To estimate the association between individual histopathological image features and survival results, we firstly divided patients into two groups according to median values of each feature. The results of univariate Cox analyses showed that 163 image features were significantly predictive of OS (*p* < 0.05, Supplementary Table 1). We also presented 20 representative image features with the most significant differences (Figure 2A), which effectively separated two survival groups. Moreover, after the LASSO-Cox regression analysis, eight histopathological features (four Zernike shape features, three granularity features, and one cells intensity characteristic) were selected. More specifically, Zernike features are a series of 30 shape features based on Zernike polynomials from order 0 to order 9 (Li et al., 2009). Granularity is a texture measurement to show the matching degree between structural elements and images texture (Vincent, 2000). Intensity-Mass Displacement describes the distance between gravity center of gray-level and binary representation of cells. The Kaplan-Meier survival curves of four image features showed the significant differences between high-level and low-level features (Figure 2B). We also analyzed the relation between HPV status and image features in 44 patients (33 HPV^–^ and 7 HPV^+^) by Wilcoxon rank sum test. Then 200 features had different distributions between groups, and four most significant features were provided in Figure 2C.

### Integrative Model of Histopathological Image Features With Genomics

**FIGURE 3:**
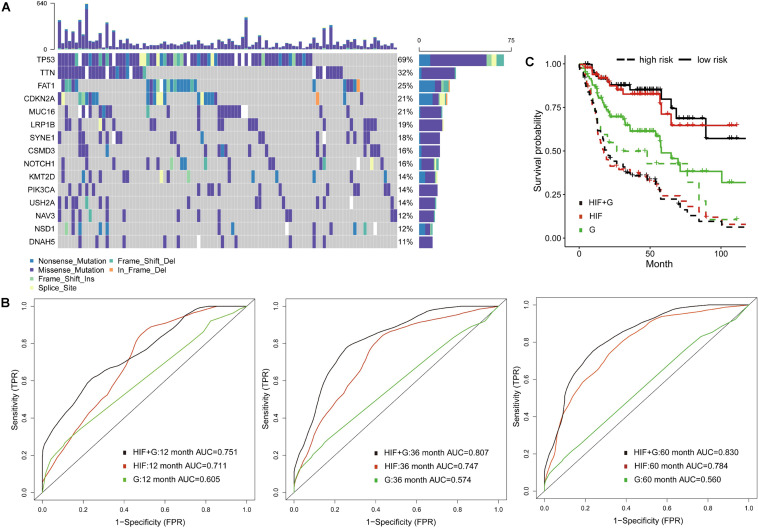
Prognostic model integrating histopathological image features with genomics. **(A)** The waterfall plot of 15 common somatic mutant genes in training set. **(B)** The 1-, 3-, and 5-year area under the time-dependent receiver operating characteristic curve (AUC), and **(C)** Kaplan-Meier survival curves showed improved predictive performance of integrative histopathology + genomics model (HIF + G) than histopathological image features alone (HIF) or genomics alone (G) in validation set.

To decrease the dimension of genomics data and increase stability of analyses, we examined the gene mutation status in the training set, and involved 100 most common somatic mutations in prognostic models (Supplementary Table 2). The waterfall plot showed 15 most frequently altered genes (Figure 3A). Previous studies also reported frequent mutations of TP53, CDKN2A, PIK3CA, NOTCH1, and NSD1 in HNSCC (Huang et al., 2019). Among them, the tumor suppressor protein p53 (TP53) mutation is commonly detected in HNSCC with report rate of 50–80% (Poeta et al., 2007), which can inhibit regulatory function of cell cycle, DNA repair and apoptosis (Vogelstein et al., 2000). In addition, mutations of TP53, p16INK4a, and overexpression of cyclin D1 and MET were regarded as poor predictors of survival and cancer progression in HNSCC patients (Bova et al., 1999; Muzio et al., 2006; Poeta et al., 2007).

Compared with traditional ROC, the time-dependent ROC is more suitable for time-to-event outcome and can comprehensively describe the predictive models (Kamarudin et al., 2017). In the validation set, we found that histopathological image features (HIF) model reached better AUCs of 1-year (0.711 vs. 0.605), 3-year (0.747 vs. 0.574), and 5-year (0.784 vs. 0.560) than genomics model (G). Furthermore, the model (HIF + G) including image features and genomics mutations had improved predictive accuracy (AUC = 0.751, 0.807, 0.830) than models using HIF or genomics alone (Figure 3B). Afterward, we divided patients into high-risk and low-risk groups by median value of risk score predicted from each model. The integrative model (HIF + G) showed better performance for prognosis (*HR* = 5.49, 95%CI: 3.17–10.90, *p* < 0.001, Figure 3C) than single-omics in HNSCC patients (Table 2).

**TABLE 2:** Predictive performance of prognostic models.

Data category	Single-omics model			Data category	Integrative model		
			
	HR	95% CI	*P*-value		HR	95% CI	*P*-value
HIF	4.77	2.51–9.06	<0.001	HIF + genomics	5.49	3.17–10.90	<0.001
Genomics	2.13	1.16–6.22	0.037	HIF + transcriptomics	6.26	2.79–9.71	<0.001
Transcriptomics	2.73	1.69–5.88	<0.001	HIF + proteomics	3.98	1.79–6.41	<0.001
Proteomics	2.33	1.26–4.31	0.007	Multi-omics model	10.66	5.06–26.8	<0.001

**HIF, histopathologic image features; HR, hazard ratio, CI, confidence interval.**

### Integrative Model of Histopathological Image Features With Transcriptomics

**FIGURE 4:**
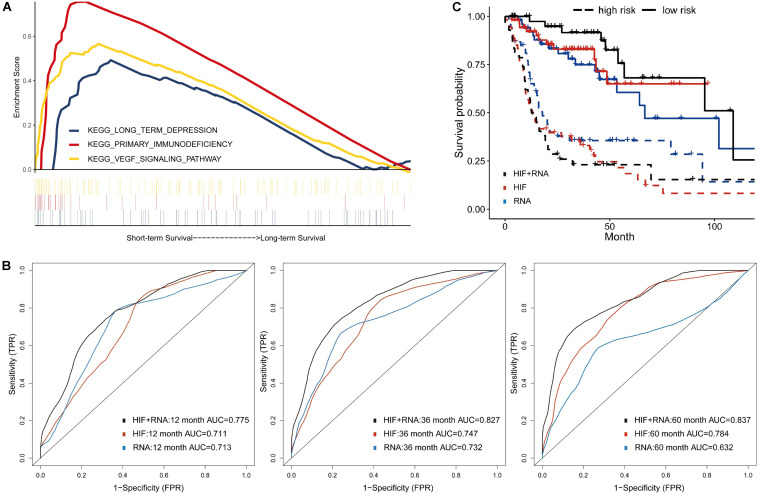
Prognostic model integrating histopathological image features with transcriptomics. **(A)** Three representative signaling pathways in short-term survival patients of training set by Gene Set Enrichment Analyses (GSEA). **(B)** The multivariate model of histopathological and transcriptomics features (HIF + RNA) reached higher 1-, 3-, and 5-year AUCs, and **(C)** more significant survival difference of Kaplan-Meier curves than models of image features alone (HIF) or transcriptomics alone (RNA) in validation set.

Besides genomics analysis, transcriptomics is also an important mean to estimate cells phenotype and function, and provides additional information of tumor features. To reduce the dimensionality, some patients of training set were classified into two groups based on survival status (12 months ≥ uncensored OS ≥ 1 month vs. OS ≥ 60 months), and 100 differently expressed mRNA genes (p_*adj*_ < 0.05) between groups were selected (Supplementary Table 3). Moreover, the GSEA of mRNA sequencing data showed that three KEGG pathways were enriched in the short-term survival group (Figure 4A). Among them, the overexpression of vascular epithelial growth factor (VEGF) can strongly induce angiogenesis in hypoxia environment of tumors, and are related with enhanced risk of death in HNSCC (Kyzas et al., 2005; Haase, 2009). Therefore, the up-regulation of VEGF signaling pathway may indicated the necessity of VEGF-targeting therapy (e.g., tyrosine kinase inhibitors).

Next, in the validation set, the transcriptomics features (RNA) yielded a good predictive performance with 1-, 3-, and 5-year AUC of 0.713, 0.732, and 0.632, which was better than genomics, but not more significant than histopathological features (Figure 4B). By combination of transcriptomics and image features, the integrative model (HIF + RNA) increased 1-year AUC to 0.775, 3-year AUC to 0.827, and 5-year AUC to 0.837. Similar results were also revealed in Kaplan-Meier survival curves (Figure 4C), the HIF + RNA model had more significant prognostic value for OS (*HR* = 6.26, 95%CI: 2.79–9.71, *p* < 0.001).

### Integrative Model of Histopathological Image Features With Proteomics

**FIGURE 5:**
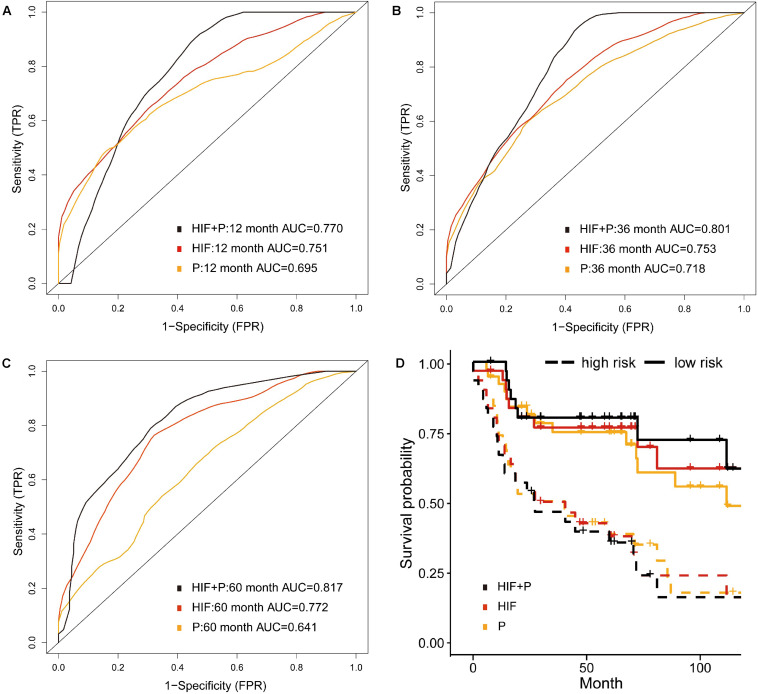
Prognostic model integrating histopathological image features with proteomics. **(A–C)** Predictive power of histopathological image features (HIF), protein expression (P), and combination of images and proteomics (HIF + P) for survival in validation set. **(D)** Kaplan-Meier curves revealed a more significant survival difference between high-risk and low-risk groups in HIF + P model.

We included proteomics data of TCPA repository via RPPA technology, which is a cost-effective method to analyze the expression and variation of marker proteins in the samples (Li et al., 2013). Totally 151 patients with protein and histopathological profiles were eligible for analyses. As shown in Figure 5C, the 5-year AUC was increased to 0.817 by incorporating image features and proteomics (HIF + P) compared with AUC of 0.772 and 0.614 for proteomics or image features alone. The 1- and 3-year AUCs were also improved when using combined features (Figures 5A,B). Moreover, the high-risk patients based on risk stratification of integrative model (HIF + P) were significantly associated with worse survival (*HR* = 3.98, 95%CI: 1.79–6.41, *p* < 0.001, Figure 5D).

### Multi-Omics Model for Survival Prediction

**FIGURE 6:**
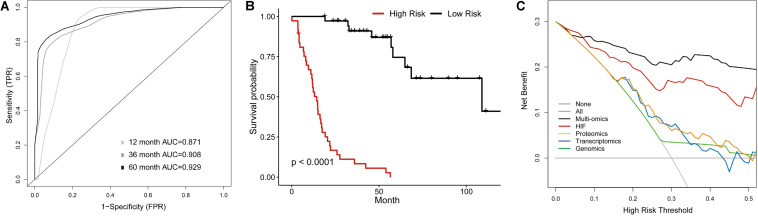
Multi-Omics Model integrating histopathological image features with omics data. **(A)** The time-dependent receiver operating characteristic curve, and **(B)** Kaplan-Meier curves for multi-omics model involving image features, genomics, transcriptomics, and proteomics in validation set. **(C)** Decision curve analysis for each model in validation set. The gray oblique line represented net benefit of intervening all patients, and horizontal gray line meant that net benefit of no patients with intervention. The multi-omics had the highest net benefit compared with other models across the range of >10% in risk threshold.

The previous analyses showed that histopathological image features had individual prognostic ability for OS. Additionally, the histopathology + omics models could improve predictive performance than genomics, transcriptomics or proteomics alone in HNSCC cohort. Finally, we established a multi-omics model to investigate the prognostic power when incorporating all above features. In the validation set, the 1-, 3-, and 5-year AUCs were 0.871, 0.908, and 0.929 (Figure 6A), which were higher than those of HIF + genomics, HIF + transcriptomics and HIF + proteomics models. Kaplan-Meier analysis demonstrated a significant different survival between high-risk and low-risk patients (Figure 6B), with a HR of 10.66 (95%CI: 5.06–26.8, *p* < 0.001). Furthermore, the multi-omics model had a better net benefit than others if the risk threshold probabilities >10% in DCA analysis (Figure 6C).

## Discussion

In this study, we extracted the histopathological image features (HIF), utilized machine-learning algorithms to establish prognostic models combining features of histopathological images, gene mutations, RNA and protein expression in training set, and estimated the prognostic capability of models in validation set of HNSCC patients. As far as we know, such finding for HNSCC is firstly reported in this research. The results showed that individual HIF were able to predict OS, especially the Zernike shape features, granularity and cells intensity. The prognostic model based on HIF reached better predictive accuracy than other omics (i.e., genomics, transcriptomics and proteomics). Moreover, the predictive performance of integrative models using more than two types of data outperformed than that of single-omics models (Table 2). The DCA curve also underlined a higher clinical net benefit of multi-omics model compared with others. Taken together, it suggested that multi-omics model integrating histopathological images with omics may be an effective risk stratification approach to improve personalized treatments in clinical practice. For instance, low-risk patients should avoid over-treatment while high-risk patients might benefit from active treatments and strict follow-up (Cheng et al., 2017).

The histopathological examination is regarded as a gold standard for diagnosis and staging in patients with cancer. However, the accuracy of grading would be affected by pathologists’ experience, and cancer patients at the same stage can have diverse survival results. The enormous amount of information in pathological slices is not easily obtained by subjective evaluation of pathologists, which poses great challenges, but also brings opportunities. Recently, the computational systems are developed to assist the image features extraction, and these features are associated with tumor characteristics and survival outcomes (Beck et al., 2011; Romo-Bucheli et al., 2016; Moon et al., 2017). The automated approach also has the strengths of improving efficiency and reducing human resource costs. Unlike previous studies including one representative or entire images (Yu et al., 2016; Cheng et al., 2017), we randomly selected 20 sub-images from whole-slide images, which decreased both computational cost and potential biases.

Additionally, our results of univariate COX analysis, multivariate LASSO and RF model all demonstrated the significant prognostic value of image features for OS. These features provide an objective and quantitative measurement of the morphology and texture of nuclei and cytoplasm. For example, the Zernike shape features mark the nucleic pixels as 1 and cytoplasmic region as 0, then produce Zernike polynomials from binary images (Li et al., 2009). Granularity estimates the size of image texture by using enlarged structure elements to match the texture (Vincent, 2000). It indicated the relation between survival outcomes and cell-level morphological structure (e.g., occupied area and shape) as well as the overall pixels characteristics of images (e.g., texture and intensity) in HNSCC cohort. Therefore, the histopathological images analysis may have potential practical value in predicting survival for HNSCC patients.

Given the heterogeneity and diversity of tumors, molecular and genetic detection are becoming routine approaches to differentiate cancer characteristics such as genotypes and phenotypes, and play a leading role in the field of precision oncology (O’Connor et al., 2008; Kather et al., 2019). Some studies have reported the improved effectiveness of prognostic models combining genomics and image features than individual models in other cancers (Cheng et al., 2017; Yu et al., 2017; Sun et al., 2018; Zhong et al., 2019). Our study had several differences from published articles. Firstly, machine-learning frameworks including LASSO and RF with 10-fold internal cross-validation can achieve more stable estimation of predictive ability. Secondly, instead of classical ROC curve that only determines the discrimination ability of markers at a fixed time point, we utilized time-dependent ROC to describe survival status in a range of time, and yielded dynamic values of AUC throughout the study (Kamarudin et al., 2017). Lastly, we estimated a variety of quantitative molecular biomarkers including somatic gene mutation, RNA sequencing data and protein expression. The prognostic role of each profiles and integration with histopathology were compared, which showed that the histopathology + omics models were better than models using one type of data, and multi-omics model achieved highest accuracy. Our results indicated the complementary effect between histopathological image features and other omics data for survival prediction. Therefore, we suggested that, when omics data were limited, histopathological images might provide effective features to improve prognostic prediction with small additional effort.

Previous studies have showed that patients with HPV^+^ HNSCC had a better prognosis and therapeutic response (Dayyani et al., 2010). The HPV status was considered as a validated molecular characteristic of HNSCC to guide the treatment strategies (Suh et al., 2014). For example, less intensive treatments are being considered for HPV^+^ oropharyngeal SCC patients (Mirghani et al., 2015). The CT radiomics features have been reported to distinguish RNA-defined HPV subtypes in HNSCC (Huang et al., 2019). The correlation between HPV status and histopathological image features is also worthy of research. In the situation of limited samples, we only showed the different distribution of image features between HPV^+^ and HPV^–^ groups. However, we hypothesized that identification of HPV status was within the ability of histopathological images analysis, which needs a comprehensive estimation in larger cohorts.

There were some limitations in this study. Firstly, we built models by 10-fold cross-validation in training set and conducted verification in another validation set to make predictive estimation as robust as possible. However, since it was difficult to find other datasets with complete information of histopathology and omics, this study was limited in one cohort and small sample size, lacking external validation. Therefore, the generalizability of current results should be considered within these limitations. Secondly, we balanced the basic clinical characteristics between two sets, but others such as complication and treatment may be potential confounding factors. Moreover, there may exist selection bias in TCGA dataset, because the representative tumor slices were more likely to be uploaded, and its typical histopathological patterns might help machine-learning model for classification (Yu et al., 2016). Nevertheless, clinicians actually examine many slices, thus the feasibility of proposed predictive models in clinical practice needs to be studied. Finally, as a retrospective study, although the integrative models showed prognostic value in our work, it requires prospective estimation by multi-center large-scale studies before routine use. In future research, other machine-learning or deep-learning methods (e.g., convolution neural networks) can be used to generate prognostic models, but the latter require massive samples for training (Mobadersany et al., 2018). Similarly, multiparameter such as immunochemical stained images could provide richer feature sets for predicting survival.

## Conclusion

The results indicated that histopathological image features had potential as significant prognostic biomarkers for overall survival in patients with HNSCC. The integrative models of genomics, transcriptomics, and proteomics along with histopathological image features may more accurately predict survival outcome than single-omics models, which might contribute to the risk stratification and personalized treatment for cancer patients.

## Data Availability Statement

Publicly available datasets were analyzed in this study. This data can be found here: the TCGA repository (https://portal.gdc.cancer.gov).

## Author Contributions

XM and HZ contributed to the conception and design of the work. HZ and LC performed the data analysis, interpretation, and manuscript drafting. YH and YL contributed to the data acquisition. All authors revised the manuscript, approved the submitted version, and agreed to be accountable for all aspects of the work.

## Conflict of Interest

The authors declare that the research was conducted in the absence of any commercial or financial relationships that could be construed as a potential conflict of interest.

## Footnotes

1 https://portal.gdc.cancer.gov

2 http://tcpaportal.org/tcpa/

3 http://www.cancerimagingarchive.net/

4 https://cellprofiler.org/

## Supplementary Material

Supplementary Table 1 Univariate Cox analysis of image features for two patient groups.

Supplementary Table 2 Included mutant genes.

Supplementary Table 3 Differently expressed mRNA genes.
